# Editorial: Microbiota and asthma

**DOI:** 10.3389/falgy.2023.1297425

**Published:** 2023-11-14

**Authors:** Brandon Bautista-Becerril, Kurtis F. Budden, Ramcés Falfán-Valencia

**Affiliations:** ^1^ HLA Laboratory, Instituto Nacional de Enfermedades Respiratorias Ismael Cosio Villegas, Mexico City, Mexico; ^2^ Hunter Medical Research Institute, New Lambton Heights and The University of Newcastle, Callaghan, NSW, Australia

**Keywords:** microbiota, asthma, microbiome, inflammation, exacerbation

**Editorial on the Research Topic** Microbiota and asthma

The human microbiome has emerged as a critical determinant of human health, mainly due to the multiple complementary mechanisms by which microbes tune and train host immunity. Microbiomes, particularly those in early life, are shaped by extrinsic and intrinsic factors, including many exposures that influence asthma risk (1). In this Research Topic, we present several emerging pieces of research that expand our knowledge of the role of microbiomes in asthma.

Severe eosinophilic asthma (SEA) is recognized as one of the most common severe asthma phenotypes. Post-hoc analysis from a clinical trial involving benralizumab demonstrated its increased efficacy on asthma outcomes in patients with comorbid SEA (2).

D'Amato et al. analyzed data from 205 patients with SEA receiving Benralizumab treatment recruited in the Italian ANANKE multicenter observational retrospective cohort study from 2019 to 2021 from 201 Italian hospital centers. Benralizumab administration reduced eosinophil count, annualized asthma exacerbation rate and use of oral corticosteroids, and improved asthma control and lung function in patients with and without the presence of chronic rhinosinusitis with nasal polyps.

In severe refractory acute asthma, some guidelines recommend add-on intravenous infusion and/or nebulized magnesium sulfate (MgSO_4_) (3). Nevertheless, in contrast to the standard treatment of asthma exacerbations, the clinical effect of MgSO_4_ has been much debated.

Rovsing et al. performed a systematic review and meta-analysis to provide an update on the efficacy of MgSO_4_ in asthma exacerbations in adults refractory to standard treatment. The study included 17 randomized controlled trials, eight evaluated treatments with intravenous MgSO_4_, eight with nebulized MgSO_4_, and one with intravenous and nebulized MgSO_4_. MgSO_4_ has a beneficial effect on lung function and reduces the admission rate in patients with acute asthma; none of the studies identified serious side effects. Therefore, it could be used as a last resort in patients with refractory symptoms after standard treatment.

The microbiota in the bronchial tree is characteristic and is disturbed in asthma subjects’ airways. The structure of the bacterial community in the airway might differ between different asthma phenotypes.

Cardenas et al. aimed to study age-related microbiome changes and their relationship with wheezing in asthma by amplicon sequencing of the bacterial 16S rRNA gene in oropharyngeal samples from 225 subjects aged 7 to 24 months (91 cases with recurrent episodic wheezing and 134 controls) in a rural district of Ecuador. The authors concluded that the upper respiratory tract microbiome differs between groups, with significantly increased *Streptococcus* and decreased *Veillonella dispar* and *Prevotella* in cases compared to controls. Likewise, microbiome changes could influence asthma development pathophysiology later in life and could contribute to chronic inflammation in the airway mucosa.

On the other hand, Ramos-Tapia et al. compared the variations of oral and nasal microbiota in terms of composition, structure, and function between healthy and asthmatic children from Santiago de Chile, sequencing the 16S rRNA amplicon of the nasal and oral mucosa of 63 asthmatic children and 89 healthy children. The oral microbiota diversity did not show significant differences in any diversity index evaluated, indicating no significant variation between asthmatic subjects and healthy individuals. However, the genera *Moraxella*, *Dolosigranulum*, *Haemophilus*, *Corynebacterium*, *Streptococcus*, and *Staphylococcus* dominated most asthmatic nasal mucosa samples.

The present Research Topic contributes novel information to better understand the implementation of biological and pharmacological treatments in asthma, as well as microbiome participation in asthma pathogenesis, particularly during early life exposures.
